# Comparison of DeltaScan^®^ and CAM-ICU for the Assessment of Postoperative Delirium in Patients Undergoing Cardiac Surgery and Cardiac Interventions: A Prospective Observational Pilot Study

**DOI:** 10.3390/life15101640

**Published:** 2025-10-21

**Authors:** Astrid Bergmann, Janis Fliegenschmidt, Silvia Ruggeri, Nikolai Hulde, Catharina Middeke, Claudia Bunge, Maria Preising, Carsten Hermes, Vera von Dossow

**Affiliations:** 1 Department of Anaesthesiology, Heart- and Diabetes-Center Bad Oeynhausen, Ruhr-University Bochum, 44801 Bochum, Germany; jfliegenschmidt@hdz-nrw.de (J.F.); sruggeri@hdz-nrw.de (S.R.); nhulde@hdz-nrw.de (N.H.); cmiddeke@hdz-nrw.de (C.M.); cbunge@hdz-nrw.de (C.B.); vvondossow@hdz-nrw.de (V.v.D.); 2 Department of Anaesthesiology, Heart- and Diabetes-Center Bad Oeynhausen, Medical Center East Westphalia-Lippe, University Bielefeld, 33615 Bielefeld, Germany; 3 Clinic of Anaesthesiology, University-Hospital Göttingen, 37075 Göttingen, Germany; m.preising@posteo.de; 4 Department of Nursing and Management, Hamburg University of Applied Sciences (HAW Hamburg), 20999 Hamburg, Germany; hermesbonn@googlemail.com; 5 Department of Advanced Clinical Nursing, Akkon University of Human Sciences, 12099 Berlin, Germany

**Keywords:** postoperative delirium, cardiac surgery and intervention, polymorphic waves, electroencephalography

## Abstract

Background: Postoperative delirium is frequent among elderly patients and often presents after major surgery or intervention. Validated screening tools should be routinely used to recognise this psychopathological syndrome. Delirium might be associated with systematic changes in brain network organisation, including reduced EEG connectivity. Methods: This observational single-centre pilot study aimed to evaluate the agreement between DeltaScan^®^ (Prolira BV, Utrecht, The Netherlands) and CAM-ICU screening in detecting postoperative delirium in patients undergoing cardiac surgery or cardiac intervention. Results: 1. Patients showing delirium have DeltaScan^®^ scores ≥ 3. 2. Patients scoring ≥ 3 in the DeltaScan^®^ examination do not necessarily show signs of delirium in CAM-ICU testing. 3. All patients showing delirium in CAM-ICU testing have pathological clock-drawing results. Conclusions: DeltaScan^®^ reliably detects delta wave abnormalities associated with delirium, though some patients with increased delta activity did not exhibit clinically manifest delirium.

## 1. Introduction

Postoperative delirium is a neurocognitive disorder characterised by acute changes in cognition and awareness. The syndromic presentation occurs due to underlying acute encephalopathy [1]. Postoperative delirium is frequently observed among elderly patients [2], especially after high-risk surgery such as cardiac, major orthopaedic [3], or general surgery [4]. Postoperative delirium has detrimental consequences for patients as it increases the incidence of prolonged length of stay in an intensive care unit, the overall length of stay in the hospital, and 30-day-mortality [5,6]. There are high chances that the patients’ quality of life will not return to that prior to surgery [7], and they will be discharged to places other than home [8]. As a result, postoperative delirium poses a substantial socioeconomic burden [9,10].

The Diagnostic and Statistical Manual of Mental Disorders (DSM-5) [1] provides the gold standard criteria for diagnosing delirium. However, as these criteria are impractical for routine use in acute care settings, several validated screening tools, such as the Confusion Assessment Method for Intermediate Care Medicine (CAM-IMC) [11,12,13], have been developed for clinical practice. Validated screening tools are essential and should be routinely implemented in the perioperative setting as delirium is often overlooked without structured assessments. However, both the DSM-5 criteria and screening tools focus on identifying the syndromic presentation of delirium. This presents significant challenges, as demonstrated by Numan et al. [14], who reported an interrater variability of 21% in expert evaluations using the Delirium Rating Scale Revised 1998 Edition (DRS-R-98) scoring system.

In addition to recognising the psychopathological syndrome of delirium, current research has also revolved around biomarkers of delirium. Several proteins serving as markers for inflammation or cellular destruction of the central nervous system have been identified to be potentially elevated in patients with clinically manifest delirium [15,16]. Due to low specificity, no single biomarker or organ-based prognostic tools that can serve as a diagnostic marker of delirium have been detected [17].

Sharpening the understanding of delirium pathophysiology and identifying potential risk factors are crucial to provide optimal care to the patient to prevent delirium, or at least reduce its impact. Polymorphic waves in the extended delta band of electroencephalography (EEG) tracings have been proposed as electrophysiological evidence of acute encephalopathy and are supported by several studies [18,19]. In fact, the slowing of background activity within EEG during delirium is widely described in the literature [19,20]. Previous findings suggest that EEG slowing and disrupted network efficiency are key features of delirium, supporting the use of EEG as a tool to detect acute encephalopathy [21,22].

Performing conventional EEG at the bedside is often impractical, requiring specialised personnel, time, and complex equipment. To address this challenge, a single-channel EEG device was developed to simplify the detection of polymorphic delta waves, which are key markers of acute encephalopathy and delirium. According to the findings of van der Kooi and her colleagues [19], Prolira (a privately held developer of a medical diagnostic device in Arnhem, The Netherlands) [23] have developed a single-channel EEG device to simplify the method and make it feasible for wider use. This diagnostic tool, known as the DeltaScan^®^ monitor, is used to detect polymorph delta waves.

The Shulman clock-drawing test has been described as being highly sensitive in detecting cognitive impairment in elderly patients [24]. Hence, patients showing delta waves might also score poorly in the clock-drawing test.

At the authors’ hospital, more than 6000 patients are annually submitted to cardiac surgery and cardiac intervention. As in most cardiac centres, patients above 65 years of age are the most common, representing a population with an increased risk of developing postoperative delirium. The aim of this prospective, single-centre observational pilot study is to evaluate the agreement between DeltaScan^®^, used both as a screening and scoring instrument for delta wave detection, and CAM-ICU screening in patients undergoing cardiac surgery or cardiac intervention.

## 2. Materials and Methods

Since 2020, an interprofessional and interdisciplinary team has been taking care of our patients scheduled for cardiac surgery or cardiac intervention throughout the whole procedure, from hospital admission to dismissal. In the same year, contracts were signed with German statutory health insurance providers for a multicomponent programme (German ministry of health quality contract for prevention of postoperative delirium in elderly patients (POD-QV)). The Federal Joint Committee (G-BA) in Germany introduced a programme to evaluate quality contracts between statutory health insurance providers and hospitals as a means to improve inpatient care. These contracts are evaluated by the independent Institute for Quality Assurance and Transparency in Health Care (IQTIG). Data are collected by an interdisciplinary and interprofessional team [25]. As the detection of delta waves is associated with acute encephalopathy, we decided to include the examination with DeltaScan^®^ in our assessment and compare the results. For patients to be involved in our study, their health insurance provider needed a contract with our hospital; the contract was evaluated in the programme introduced by the Federal Joint Committee, as mentioned above.

The ethics committee of Ruhr-University Bochum, Germany, approved the protocol for this study (OWL, AZ 2021-861). Data from the IQTIG-database were prospectively analysed. This study is registered in the German Clinical Trials Register (Deutsches Register Klinischer Studien) and the national WHO primary registry (DRKS00028749).

### 2.1. Subjects

The IQTIG-database was analysed, and 364 patients over 65 years of age undergoing elective cardiac surgery or cardiac intervention from May 2021 to March 2022 were included in this study. Treatment included the whole spectrum of cardiac surgical and interventional procedures, ranging from single off-pump coronary artery graft to left ventricular assist device implantation and from transfemoral aortic valve implantation to mitral clipping. Patients who were diagnosed with dementia or other neurological morbidities before admission and those with a language barrier were excluded from this pilot study. All included patients provided written informed consent. Patients undergoing surgery and cardiac intervention are included in this study because the aim is to compare two different diagnostic tools rather than searching for differences between two cohorts of patients.

### 2.2. Measurements

Patients were assessed by a well-trained team using the CAM-ICU, the Shulman clock-drawing test, and the 1-Channel-EEG (DeltaScan^®^, Lethabong, South Africa). This process was performed four times: one day preoperatively and on days one, two, and three after surgery or intervention. All three tests were carried out by the same nurse at the same time of day while patients were awake, but different nurses could have performed the exams between timepoints (one day preoperatively and days one, two, and three postoperatively). All assessors and medical staff had unlimited access to previous results as they were included in the patients’ records. All assessing nurses belong to the same well-trained interprofessional team.

Screening with the CAM-ICU was carried out according to the protocol described by Boettger et al. [26]. The CAM-ICU is considered positive, as outlined in previous publications [11,27].

The clock-drawing test yielded scores ranging from 1 to 6 according to Shulman [28,29,30], with 1 representing a perfect picture of a clock with the correct numbers and the correct times and 6 indicating that a clock is not shown at all. A score of 3 indicates that a clock with just one hand or even no hand at all is shown with the wrong times. Thus, the presence of cognitive impairment was detected with a score of 3 and above.

The assessment of DeltaScan^®^ was performed according to the manufacturer’s instructions [23,31]. It is based on an automatically analysed, one-channel EEG recording (FP2-TZ) [23]. The detection of delta waves was translated by the device into a score ranging from 1 to 5 points according to the manufacturer’s algorithm, which is not presented in detail. According to the manufacturer’s manual [23,31], the scores range from “very unlikely” (1) to “very likely” (5) regarding the detection of acute encephalopathy, with a score of 3 being labelled as “possible”, which leaves room for interpretation. In a previous publication to which the manufacturer himself refers [18], the same scores from 1 to 5 are presented, but scores 1 and 2 represent “no acute encephalopathy”, while scores 3, 4, and 5 represent “acute encephalopathy.” Ditzel et al. do not interpret score 3 as uncertain but as an indication of acute encephalopathy; thus, we adopted this interpretation in this study. DeltaScan^®^ achieved a Medical Device Regulation certification in 2020, and it is also registered as a medical device under Germany’s Medical Devices Act (Medizinproduktegesetz, MPG).

The collected data were saved along with the patients’ characteristics (age, gender, height, weight, diagnosis, and surgery or intervention), which were derived from electronic records. All measurements were stored in individual patient files, and the study data were stored separately. The personnel performing the tests were not blinded to the results.

### 2.3. Statistical Analysis

No a priori sample size calculation was performed due to the observational nature of this pilot study. Statistical analysis was conducted with R (R version 4.1.2) [32]. A total of 364 patients met the inclusion criteria, and after excluding patients with incomplete records (dropouts), 335 remained for statistical analysis.

Univariate inter-group differences for ordinally scaled variables were assessed using the Mann–Whitney U (MWU) test. Correlations between ordinally scaled measures were evaluated using Spearman’s rank correlation coefficient, and a significance level of *p* < 0.05 was applied for all statistical tests. All graphs were computed using ggplot2 [33] in R version 4.1.2. Where displayed, regression analysis was performed with a linear regression model.

A post hoc power analysis was performed using the pwr package in R to determine the achieved statistical power for our diagnostic accuracy metrics. For sensitivity, the achieved power was >99.9%. For specificity, the achieved power was 94.9%. These results, calculated with a significance level (α) of 0.05, demonstrate that the study was adequately powered to detect the reported accuracy of the diagnostic tool. The correlation tests between postoperative and DeltaScan^®^ readings in the perioperative period were powered sufficiently as well with all power calculations exceeding 0.99 at a 0.05 significance level.

## 3. Results

A total of 364 patients were included in this study, of whom 335 were analysed after excluding cases with incomplete data. Upon further investigation, 14 patients were excluded due to not wanting to participate in the study (they did not provide consent). For the other patients who were excluded, it was not always clear why they were not marked as completed. We suspect that this could be due to RASS-5 sedation during the entire follow-up period, for example, but reasons were not explicitly documented for all cases. Demographic data are shown in Table 1. A total of 50 patients (14.9%) showed delirium postoperatively according to the CAM-ICU. Patients were considered to be experiencing delirium if any of the three postoperative CAM-ICU tests were positive. Due to the short follow-up timeframe, the fluctuating nature of delirium, and the close association with the intervention, episodes within affected patients were not further differentiated.

**Figure 1:**
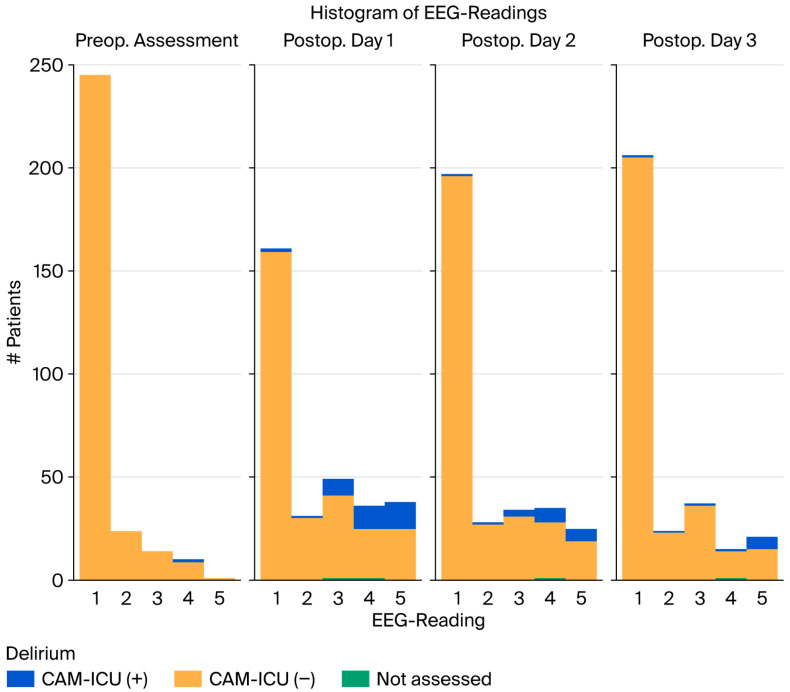
Column plot representing the scores in the CAM-ICU and DeltaScan^®^ preoperatively and on days 1 to 3 postoperatively.

A DeltaScan^®^ score ≥ 3 was significantly associated with postoperative delirium (*p* < 0.01, Mann–Whitney U test), as presented in Figure 1. The yellow columns represent patients without clinical signs of delirium, whereas the blue columns represent patients showing the clinical picture of delirium according to the CAM-ICU. It can be seen that patients with delirium also have DeltaScan^®^ scores ≥ 3. However, not all patients scoring ≥ 3 in DeltaScan^®^ show signs of delirium in CAM-ICU testing.

On postoperative days 2 and 3, the number of patients showing postoperative delirium according to the CAM-ICU decreased, and the number of those without postoperative delirium increased. Still, some patients scored ≥ 3 in DeltaScan^®^ without being diagnosed with delirium after CAM-ICU testing, and patients diagnosed with delirium all had scores ≥ 3 in DeltaScan^®^.

**Figure 2:**
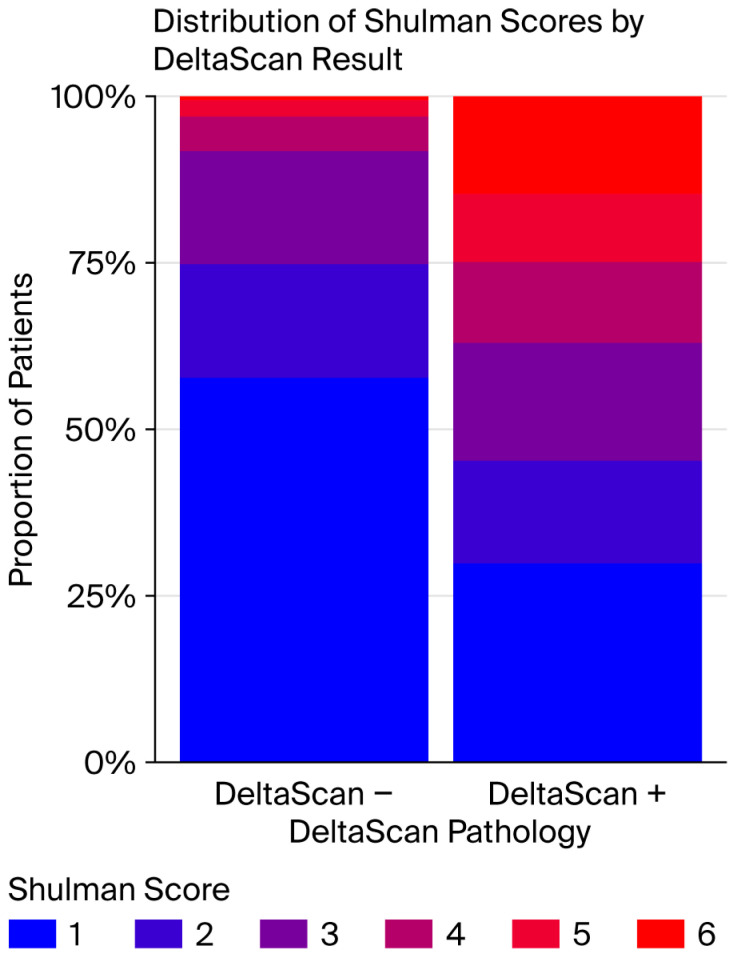
Distribution of Shulman test scores for all postoperatively gathered DeltaScan–Shulman score pairs.

Patients with DeltaScan^®^ scores ≥ 3 also showed scores ≥ 3 in the clock-drawing test, as illustrated in Figure 2. The column on the left represents patients with DeltaScan^®^ scores < 3, with 75% of them scoring 1 and 2 in the Shulman test. The right column shows patients with DeltaScan^®^ scores ≥ 3, indicating that more than 50% of them scored ≥ 3 in the Shulman test. In other words, patients showing polymorph delta waves drew poorer pictures in the clock-drawing test, and patients without polymorph delta waves drew the clocks more accurately.

**Figure 3:**
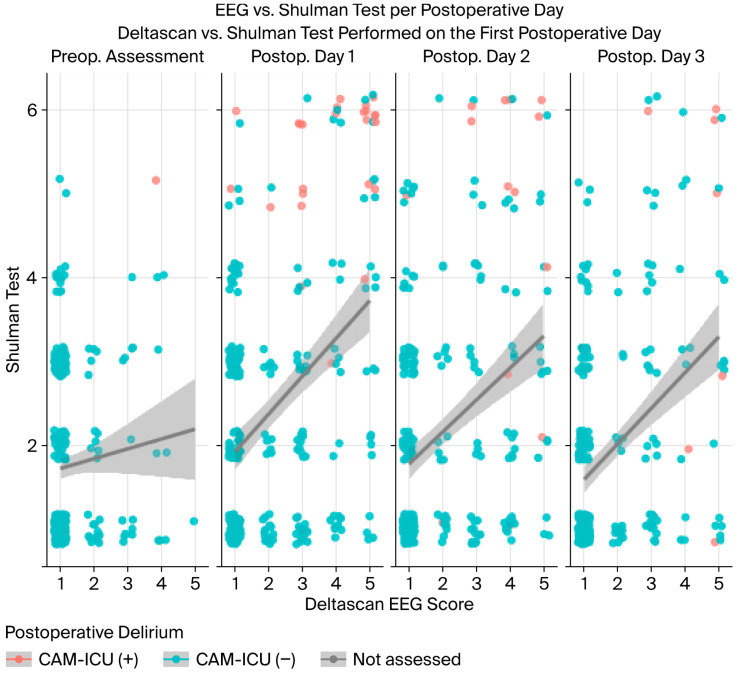
Dot diagram showing the scores of the clock-drawing test in different score groups of DeltaScan^®^.

Furthermore, all patients diagnosed with delirium based on the CAM-ICU had clock-drawing test scores ≥ 3, as illustrated in Figure 3. Patients without delirium according to the CAM-ICU are represented by blue dots, while those with delirium are represented by orange ones. Again, it can be demonstrated that patients with delirium tend to score ≥ 3 both in DeltaScan^®^ and the Shulman test, indicating poorer clock-drawing skills and displaying polymorph delta waves in the presence of delirium. Still, not all patients scoring ≥ 3 in DeltaScan^®^ show clinical signs of delirium, as shown in Figure 1.

## 4. Discussion

The main results of the present observational study are as follows: (I) Patients showing delirium (CAM-ICU positive) had DeltaScan^®^ scores ≥ 3, i.e., clinically manifest postoperative delirium was strongly associated with the presence of delta wave abnormality. (II) Patients scoring ≥ 3 in the DeltaScan^®^ examination did not necessarily show signs of delirium in CAM-ICU testing, i.e., some patients with delta wave abnormality did not exhibit clinically manifest delirium. In addition, (III) patients showing delirium in CAM-ICU testing all had pathological clock-drawing results, i.e., delirium was strongly related to impaired cognitive ability.

Our findings are in line with previous publications demonstrating that DeltaScan^®^ results were similar to those obtained from other scoring systems like the CAM-ICU [34], the delirium rating scale revised from 1998 (DRE-R-98) [35], or the delirium observation scale (DOS) [36]. We obtained our data from a large database that only includes patients undergoing cardiac surgery or cardiac intervention. Therefore, this is the first study to describe delirium in this highly vulnerable cohort with a considerable number of patients. In a previous publication [19], similar results were shown in cardiothoracic patients, but that cohort is significantly smaller than the present one, with only 28 patients. We can confirm that DeltaScan^®^ could be a useful tool in clinical practice to identify the presence of polymorphic delta waves as markers of acute encephalopathy in patients undergoing cardiac surgery and interventions.

The fact that patients achieved scores ≥ 3 in DeltaScan^®^ testing without showing clinical signs of delirium or acute encephalopathy supports the hypothesis that DeltaScan^®^ is a useful tool for the early detection of delirium [34,35,36]. This aligns with our findings, as DeltaScan^®^ detects polymorphic delta wave activity, which may reflect the breakdown of functional brain networks described in these models. Given that acute encephalopathy is associated with reduced EEG connectivity and decreased network efficiency, DeltaScan^®^’s ability to detect delta wave abnormalities might make it a valuable tool to indicate early functional disintegration. Furthermore, these findings suggest that EEG-based monitoring could identify patients at risk for delirium before clinical symptoms become apparent.

Frailty and postoperative delirium were assessed in a previous publication [25] from our centre. Assessing cognitive impairment using the clock-drawing test according to Shulman [29] is significant as this test yielded a result for every patient in all four timepoints in which the exams were performed. When the phenotypes of delirium or acute encephalopathy, as diagnosed using DeltaScan^®^, were compared to the results of cognitive impairment diagnosed using the Shulman test, there was a large congruence between tests. This supports the hypothesis that the detection of delta waves in patients that are not showing signs of delirium is an indication that delirium may develop later. This state manifests itself through the pathological clock drawing. It might be postulated that the presence of polymorphic delta waves reveals cognitive impairment that eventually leads to clinically manifest delirium. To further assess this hypothesis, trials are needed to analyse whether patients showing delta waves eventually develop delirium and whether this can be prevented by early treatment. Due to the observational nature of this pilot study, this question has not been posed in this context. A standard operating procedure (SOP) is available for the treatment of postoperative delirium in our centre [25]. It includes the CAM-ICU test as a screening instrument for delirium, additional pain and sedation scores, standards for preoperative treatment such as the avoidance of benzodiazepines as premedication, and guideline-based recommendations for non-pharmacological delirium prevention in the postoperative phase. These represent the most important measures: maintaining the circadian rhythm, communication with relatives, and optimising environmental conditions (noise, light, orientation aids). Pharmacotherapy is also presented in terms of indication, goal, and medication. In addition, the implementation of ultra-fast track concepts is supported by the use of short-acting intravenous anaesthetic agents intraoperatively and the use of dexmedetomidine. The SOP is a local implementation of the delirium guidelines of the German Association of Anaesthesia and Intensive Care (DGAI) and the European Society of Anaesthesiology and Intensive Care (ESAIC) [37,38].

Jones and his colleagues conducted a comprehensive review of publications on delirium assessment tools and scoring systems [39], and subsequently outlined the characteristics of an ideal instrument. DeltaScan^®^ is easy to apply and only needs little training before use, it can be used on non-responsive patients—i.e., intubated patients in the ICU—and it delivers results that are not dependent on the examiner. This shows that DeltaScan^®^ meets the criteria for an ideal assessment tool. In addition, patients displaying delta waves without showing signs of delirium are identified, which might allow for early intervention and treatment. The fact that patients showing delta waves have a pathological result in the clock-drawing test—hinting at cognitive impairment—further supports this. In addition, this tool can easily be integrated into the clinical workflow and is highly accepted among nursing staff.

Our study has some limitations that need to be addressed.

Both cardiac surgery and intervention patients are included. The present study aimed to find a possible agreement between the detection of polymorphic delta waves using DeltaScan^®^ and diagnosing delirium using the CAM-ICU, thus focusing on two diagnostic tools rather than on two different cohorts of patients.

In addition, the patients included in this study were very heterogeneous in terms of underlying diseases, comorbidities, and clinical treatment. This led to differences in the duration of mechanical ventilation and the length of stay in the ICU and hospital. Still, we aimed to compare two technical tools, DeltaScan^®^ and the CAM-ICU, to detect delirium. Future research in randomised controlled trials is needed to evaluate the use of DeltaScan^®^ in homogenic patient cohorts to exclude confounders such as comorbidities and surgical or interventional impact.

The fact that the assessors performing the different tests were not blinded to the results of the patients’ previous examinations might have influenced the interpretation of the CAM-ICU results. Therefore, following the present pilot study, more blinded randomised controlled trials are needed to reduce the risk of measurement and selection bias.

The present study is a monocentric one as it only includes a limited patient cohort. The centre where the data were collected is a specialised heart centre that treats patients all over the country and is therefore not geographically limited. Thus, the advantages of a single-centre study—namely, reduced costs, lower organisational effort, and easier coordination and data acquisition—outweighed the disadvantages.

Next, the scoring system of DeltaScan^®^ is inconsistent; according to the manufacturer [23,31], a score of 3 represents uncertainty in the detection of acute encephalopathy as it is labelled “possibly.” However, the manufacturer refers to a publication in which a clear cut-off is drawn, with scores of 1 and 2 representing “no acute encephalopathy” and scores of 3 to 5 representing “acute encephalopathy” [18]. According to Ditzel et al., sensitivity and specificity were high when using this cut-off, so it was also used in this study. Regardless, a score of 3 in DeltaScan^®^ testing might be confusing as patients showing delirium achieve scores ≥ 3, but not all patients with a score of 3 show delirium. Future research through blinded randomised controlled trials is needed to assess the score of 3 in DeltaScan^®^ in more detail.

This study evaluated the diagnostic value of DeltaScan^®^ compared to the CAM-ICU and the clock-drawing test in a cohort of patients undergoing cardiac surgery or interventions. The findings confirm that DeltaScan^®^ reliably detects delta wave abnormalities associated with delirium, though some patients with increased delta wave activity did not exhibit clinically manifest delirium. Therefore, DeltaScan^®^ might grant early insights into the future development of delirium. Whether the clinical picture of delirium can be prevented in patients showing polymorphic delta waves through timely intervention was not assessed in this pilot study, but should be analysed in future studies. The CAM-ICU should not be replaced as the standard method for detecting delirium. In cases where the CAM-ICU is difficult to perform—for example, in non-cooperative patients—DeltaScan^®^ can be used as an additional screening tool with minimal barriers to application. This tool might hint at developing conditions, improve accuracy in diagnosis, and eventually increase the quality of patient care and outcomes. However, this needs to be evaluated in future blinded randomised controlled trials.

## Figures and Tables

**Table 1:** Demographic data of the cohort. (Cath: catheter intervention, POD: postoperative delirium, Surg: surgical).

Variable	Overall N = 335	POD − N = 285	POD + N = 50
Age	77 (7)	77 (7)	78 (6)
Sex			
M	179/335 (53%)	154/285 (54%)	25/50 (50%)
F	156/335 (47%)	131/285 (46%)	25/50 (50%)
Intervention			
Surg	183/335 (55%)	141/285 (49%)	42/50 (84%)
Cath	152/335 (45%)	144/285 (51%)	8/50 (16%)

## Data Availability

The data presented in this study are available on request from the corresponding author due to local legislation and privacy regulations. Data can be made available only after signing a collaboration agreement.

## Abbreviations

The following abbreviations are used in this manuscript:

CAM-ICU	Confusion Assessment Method for Intermediate Care Medicine
DSM-5	Diagnostic and Statistical Manual of Mental Disorders
DRS-R-98	Delirium Rating Scale Revised 1998 Edition
EEG	Electroencephalography
fMRI	Functional Magnetic Resonance Imaging
POD-QV	Postopeatives Delirium-Qualitätsvertrag
G-BA	Gemeinsamer Bundesausschuss
IQTIG	Institut für Qualität und Transparenz im Gesundheitswesenelectroencephalography
DRKS	Deutsches Register Klinischer Studien
